# Clinical association and diagnostic significance of miRNA-29a and miRNA-147b in type 2 diabetes mellitus

**DOI:** 10.7150/ijms.84899

**Published:** 2023-08-28

**Authors:** Phan The Dzung, Ngo Tat Trung, Le Van Khanh, Duong Dinh Chinh, Doan Van De, Hoang Van Tong, Nguyen Linh Toan

**Affiliations:** 1 Endocrine Hospital, Nghe An, Vietnam.; 2 Department of Pathophysiology, Vietnam Military Medical University, Hanoi, Vietnam.; 3 Centre for Genetics Counsulation and Cancer Screening, 108 Military Central Hospital, Hanoi, Vietnam.; 4 Institute of Biomedicine and Pharmacy, Vietnam Military Medical University, Hanoi, Vietnam.; 5 Internal Department, Vinh Medical University, Nghe An, Vietnam.; 6 103 Military Hospital, Vietnam Military Medical University, Hanoi, Vietnam.

**Keywords:** miR-29a, miR-146a, miR-147b, Type 2 diabetes mellitus, Beta-cell dysfunction

## Abstract

**Background:** Micro RNAs (miRs) expression is involved in the pathogenesis of type 2 diabetes mellitus (T2DM). This study investigates the expression levels of plasma miR-29a, miR-146a, and miR-147b and their correlations with clinical parameters in patients with T2DM.

**Methods:** 105 patients with T2DM who categorized either as newly diagnosed T2DM (n=52) or treated T2DM (n=53) and 93 healthy individuals were included in this study. The expression levels of miR-29a, miR-146a, and miR-147b were quantified by real-time PCR and analyzed for possible association with T2DM.

**Results:** The expressions of miR-29a and miR-147b were significantly increased in T2DM patients compared with healthy controls (*P*<0.0001). The expression levels of miR-29a in newly diagnosed T2DM patients were higher than that in the group of treated T2DM (*P*=0.002). The expression of studied miRs was correlated with several clinical parameters such as blood glucose levels, HbA1C, microalbuminuria, C-peptide, triglyceride levels as well as the HOMA-β index. The expression levels of miR-29a and miR-147b show a potential diagnostic performance to discriminate newly diagnostic T2DM (AUCs=0.77 and 0.84, respectively) and beta-cell dysfunction (AUCs= 0.62 and 0.75, respectively).

**Conclusions:** The plasma miR-29a and miR-147b expression levels in T2DM patients are significantly associated with T2DM while miR-146a shows poor evidence in relation to T2DM. miR-147b shows potential as a biomarker for the diagnosis of T2DM and pancreatic beta cell dysfunction.

## Introduction

Type 2 diabetes mellitus (T2DM) is a chronic disease characterized by hyperglycemia associated with abnormalities in carbohydrate, lipid, and protein metabolism. According to the latest statistics from the International Diabetes Federation (IDF) in 2021, there were about 537 million people (aged 20-79 years) with diabetes mellitus in the world 1. This number is predicted to increase to 643 million in 2030 and 783 million in 2045. The highest rate of increase tends to shift from countries with developed economies to poor and developing countries. Diabetes mellitus is the fourth leading cause of death in the world and shortens life expectancy by 5 to 10 years. The cost of treating diabetes mellitus worldwide is estimated at $760 billion in 2019. In Vietnam, as of 2012, the prevalence rate of diabetes mellitus in adults is 5.7%; the pre-diabetes rate is 12.8%. T2DM constitutes up to 95% of all diabetes and is characterized by chronic hyperglycemia due to the disorders of insulin secretion and/or insulin action and impaired lipid and protein metabolism. The pathogenesis of T2DM is due to insulin resistance, impaired beta cell function, and the loss of the body's ability to regulate blood glucose levels 2. Micro RNA (miR) expression is associated with regeneration, regulation of beta cell function 3 or programmed beta cell death 4, and insulin resistance 5.

In pancreatic cells, high glucose levels stimulate mitochondrial oxidation, increase the intracellular ATP/ADP ratio, and thereby promote insulin secretion by beta cells. The gene that regulates this process is monocarboxylate transporter-1 (Mct1). Therefore, abnormal expression of the *MCT1* gene affects insulin secretion. Three isoforms of miR-29, including miR-29a, miR-29b, and miR-29c were highly expressed in pancreatic beta cells 6. The miR-29 acts on the promoter region of the *Mct1* gene and inhibits the expression of the *MCT1* gene. This inhibits insulin secretion by pancreatic beta cells. MiR-29 overexpression also promotes programmed cell death 7. In contrast, miR-29a depletion improved beta cell function 8. Thus, miR-29 plays an important role in beta cell dysfunction in diabetes mellitus. Furthermore, there was an increase in the expression of miR-146a and miR-34a in MIN6 beta cell lines when exposed to esters for 3 days and causing decreased insulin secretion, increasing beta cell death from the program 9.

Insulin resistance is important pathogenesis of T2DM in which the insulin signaling pathway plays a central role. There is evidence that insulin resistance is associated with insulin signaling deficiencies and that miR is involved in this process. Insulin-receptor interactions are the first step in insulin signaling. Mice lacking the INSR gene have hyperglycemia and hyperinsulinemia, and a large number of studies have shown decreased INSR in patients with type 2 diabetes. The miRs bind directly to the 3-UTR end of the INSR gene, resulting in impaired insulin signaling in hepatocytes 10. In adipocyte cells, miR-29a expression increases and inhibits IRS-1 gene expression by directly acting on the 3-UTR region of the IRS-1 gene 11. Several miRs indirectly inhibit the expression of the IRS-1 gene 5. Furthermore, the upregulation of miR-29 led to the suppression of glucose uptake in rat adipose tissue and 3T3-L1 adipocytes 12. Thus, increased miR-29 expression could contribute to the pathogenesis of T2DM.

Recently, the role of miR-146a has also been investigated for pro-inflammatory factors in glycemic control and insulin resistance. MiR-146a significantly reduced expression in peripheral blood mononuclear cells. miR-146a expression is negatively correlated with HbA1C, TRAF6 mRNA, TNF, IL-6 and insulin resistance. Reduced miR-146a expression impacts poor glycemic control, insulin resistance, genes of some pro-inflammatory factors, and blood levels of INF and IL-6 in patients with T2DM 13. Although miR-147b has been shown to circulate in peripheral blood samples, there are only a few studies on miR-147b 14. In a rat model of obesity with diabetes mellitus, an increase in miR-147 overactivated the inflammatory property of macrophages, enhancing the inflammatory process that helps promote periodontitis in diabetes mellitus rats 15. The expression of miR-147b is activated by lipopolysaccharides and hypoxia induces cellular effects such as proliferation, migration, and apoptosis 16. This study aimed to investigate the expression levels of plasma-free miR-29a, miR-146a and miR-147b and to analyze the association with some clinical and subclinical features, insulin resistance in Vietnamese patients with T2DM.

## Materials and Methods

### Patients and controls

The study was conducted on 198 subjects who were divided into two groups: the disease group consisted of 105 patients diagnosed with T2DM, and the control group consisted of 93 people without diabetes. Based on the time of diagnosis of T2DM, patients were divided into two subgroups, including patients with newly diagnosed T2DM without interventional therapy (n = 52) and patients with T2DM with therapeutic intervention (n = 53).

Patients aged 40-70 years are diagnosed with T2DM according to the American Diabetes Association (ADA) 2014 standard. Anthropometric indices such as height, weight, waist and hip circumference were measured for all study participants. Body mass index (BMI) and waist-to-hip ratio (WHR) were calculated based on their anthropometric indices. The exclusion criteria for the disease group were undiagnosed patients with T2DM. Patients with acute and severe infections, malignancies, and diseases affecting glucose metabolism. The patient is addicted to alcohol or tobacco. The patient did not consent to participate in the study. The control group consisted of people between the ages of 40 and 70 without diabetes or prediabetes. All subjects in the control group were clinically examined and considered healthy at the time of sampling. None of them had malignancies, had severe acute infections, or was taking medications that affect glucose metabolism within a month. In addition, diabetes-related symptoms and personal and family history of diabetes; smoking; alcohol consumption was also the exclusion criterion for the control group.

### Ethics statement

Written informed consent was obtained from all study participants. The study was approved by the Institutional Review Board of Vietnam Military Medical University and the Review Board of Endocrine Hospital, Nghe An Province, Viet Nam.

### Measurement of biochemical parameters

Biochemical tests were performed on Abbott's Architect i2000SR system (USA). Glucose levels were quantified by a kinetic method involving the enzyme hexokinase. Subjects were tested for glucose tolerance at the first visit to diagnose diabetes. Insulin was quantified by electroluminescent immunoassay on an automated immunoassay machine. The determination of HbA1c in fasting blood is based on the principle of high-pressure liquid chromatography (HPLC). C-peptide was quantified by ECLIA electroluminescence. Blood lipid components including cholesterol, triglycerides, high-density lipoprotein cholesterol (HDL-C) and low-density lipoprotein cholesterol (LDL-C) were quantified on an automated biochemical machine. Microalbumin was quantified by an immunoturbidimetric method. Insulin resistance indices: HOMA-IR, HOMA-S, HOMA-β according to the HOMA-2 model using the software (https://www.dtu.ox.ac.uk/homacalculator). We used glucose and C-peptide pairs to calculate indices of insulin resistance and beta cell function.

### Quantification of miR-29a, miR-146a and miR-147b

The total RNA was extracted from the plasma samples of patients and controls using the phenol/chloroform method. cDNA is synthesized from the extracted total RNA (Reverse transcription Polymerase Chain Reaction) by using the RevertAid First Strand cDNA Synthesis Kit. After reverse transcription, cDNA was reconstituted in 100 μl 25 mM-Tris-HCl pH 8.0. The real-time PCR (qPCR) reaction mixtures consisted of 10 μl of 2x Sybr-Green I master mix (Applied Biosystems, Foster City, CA, USA), 5 μl of cDNA preparation, 5 pmol of miRNA universal reverse primer GTGCAGGGTCCGAGGT and 5 pmol of forward primer specific for miR-29a, miR-146a, and miR-147b (Table 1). The qPCR reaction was performed using the Stratagene M3000p device (Stratagene, San Diego, CA, USA) with a pre-incubation step at 50 °C for 15 minutes, initial denaturation at 95 °C for 5 minutes, followed by 45 cycles of 95 °C for 15 sec and 60 °C for 60 seconds. The real-time PCR reactions were finalized by amplicon melting dissociation. The cycle of threshold (Ct) values were recorded and analyzed according to the comparative Ct method, in which the Ct value of miR-39 was used as a normalization factor as recommended previously miR = 2^-ΔCt^ (ΔCt = Ct (target miR) - Ct (control miR)) 17.

### Statistical analysis

The variables were tested for normality using the Kolmogorov-Smirnov test. The data were calculated and presented as mean and standard deviation or median and quartile where appropriate. Two means were compared using Student's test (t-test), and more than two means were compared using the ANOVA test. Two medians were compared using Mann-Whitney nonparametric test, and multiple medians were compared using the Kruskal-Wallis test. The correlation between the variables was assessed by the correlation coefficient (r) (Pearson's correlation or Spearman's correlation where appropriate). Qualitative variables are presented as percentages (%) and the differences between qualitative variables were tested by Chi-square. The regression model was used to evaluate the linear relationship between the dependent variable and the independent variable. Assess the diagnostic value of miRs by the area under the ROC curve (AUC). The sensitivity, specificity and cutoff values were also calculated. All statistical analyzes were performed using IBM Statistics SPSS Version 22 (IBM Corp, Armonk, New York, USA) and the level of significance was set at a P-value of less than 0.05.

## Results

### Demographic, clinical and biochemical characteristics of the study participants

The demographic, clinical, and biochemical characteristics of patients with T2DM and symptomatic controls are summarized in Table 2. There was no statistical difference between the mean age and male/female ratio of the patients with T2DM (55.85 ± 8.35) and the controls (54.03 ± 8.36) (*P* > 0.05). The anthropometric indices of BMI and waist circumference of the patients were similar to those of the controls (*P* > 0.05). However, the median of the WHR index was higher in the patient group [0.92 (0.8-1.04)] in comparison to those of the control group [0.89 (0.76-1.1)] (*P* < 0.001). In addition, overweight and obesity were relatively high (53.3%) in patients with T2DM, with overweight accounting for 25.7% and obesity is of 27.6% (data not shown). Both fasting blood glucose levels and HbA1C were significantly elevated in patients with T2DM compared to controls, while were decreased in the treated T2DM. In the group of patients with treated T2DM, the percentage of patients with glycemic control who achieved target HbA1C < 7% was 50.9%. However, the C-peptide levels in the control group were higher than those in the group with newly diagnosed T2DM and treated T2DM. In addition, the proportion of patients with T2DM in the study was 62.9% with lipid metabolism disorders. The insulin resistance index (HOMA-IR) of the patient group with T2DM was higher than that of the control group (*P* < 0.05). Patients with T2DM had a significantly lower beta cell function index (HOMA-β) compared to the controls (*P* < 0.0001) (Table 2).

### Plasma miR-29A, miR-146A, and miR-147B expression levels in patients with T2DM and controls

**Figure 1:**
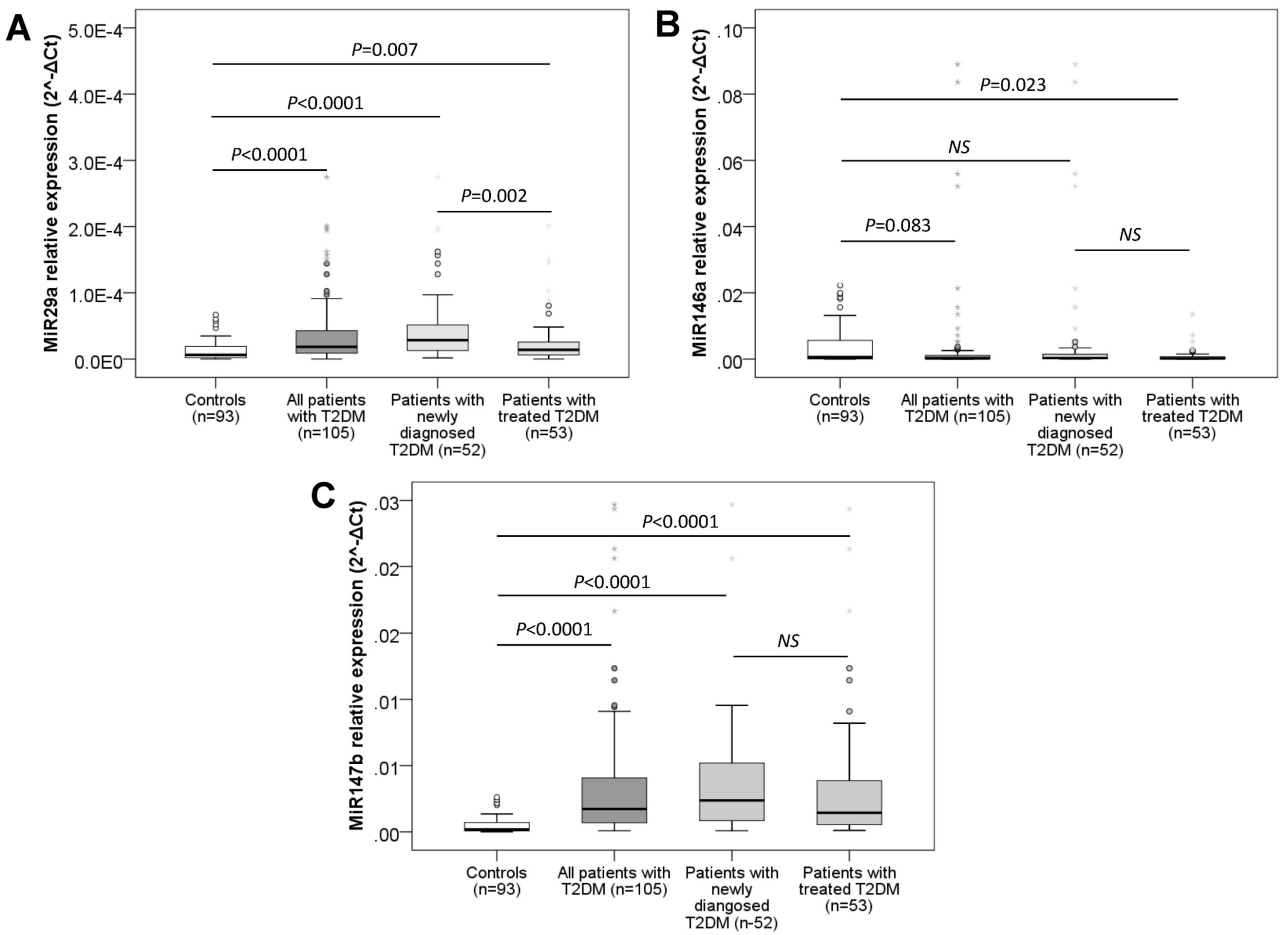
** Plasma expression levels of studied miRNAs in patients and controls.** The expression levels of plasma miR-29a (**A**), miR-146a (**B**) and miR-147b (**C**) were measured and compared between patients with T2DM, subgroups of patients and controls. P values were calculated by using Mann-Whitney tests.

The expression levels of plasma miR-29a, miR-146a and miR-147b were measured and compared between patients with T2DM, subgroups of patients and controls. We observed that the expression levels of miR-29a and miR-147b were significantly higher in patients than in the control (*P* < 0.0001) (Figure 1) while the expression level of miR-146a in T2DM was lower than the control group (*P* = 0.083) (Figure 1). When comparing the plasma miRs expression levels of the groups of newly diagnosed and untreated T2DM patients with controls, the expression levels of miR-29a and miR-147b were significantly higher in newly diagnosed T2DM patients as well as in the treated T2DM group than those in the control group (*P* = 0.007 and < 0.0001 for miR-29a; *P* < 0.0001 for miR-147b). Furthermore, we observed that the expression levels of miR-29a in newly diagnosed T2DM patients without treatment were higher than that in the group of T2DM with treatment (*P* = 0.002) while the expression of miR-146a and miR-147b between the newly diagnosed T2DM group and the treated T2DM group was not statistically different (*P* > 0.05). However, the expression level of plasma miR-146a was lower in the group of patients with T2DM receiving treatment intervention than in the control group (*P* = 0.023), while the plasma expression of miR-146a in the group of newly detected T2DM patients who had not been treated yet was not statistically different when compared to the controls and the treated T2DM group (*P* > 0.05) (Figure 1).

### Correlation of expression levels of miR-29a, miR-146a, and miR-147b with clinical parameters

We analyzed the correlation of miR-29a, miR-146a, and miR-147b expression levels with clinical characteristics of patients with T2DM and the results showed that the expression level of miR-29a had a weak negative correlation with the disease duration (r = -0.28; *P* = 0.004). The expression level of mir-29a did not correlate with other demographic parameters such as weight, height, BMI, and WRH. The expression level of miR-146a was correlated with the age of patients (r = -0.2; *P* = 0.04) but did not correlate with other clinical indicators. In addition, the expression level of miR-147b was not correlated with all clinical indicators (Table 3).

Concerning laboratory parameters, the expression level of mi-29a in T2DM is positively correlated with blood glucose levels and HbA1c, inversely correlated with the beta cell functions (HOMA-β). The expression level of miR-29a correlated positively with the expression of miR-146a (r = 0.2; *P* = 0.045) and moderately positively with the expression of miR-147b (r = 0.47; *P* < 0.001). However, levels of insulin, C-peptides, blood lipids, microalbuminuria, HOMA-IR, and HOMA-S did not correlate with the expression level of miR-29a (Table 4). In the group of newly diagnosed T2DM patients who received no treatment, we found a correlation between miR-29a and miR-147b expressions (r = 0.45; *P* = 0.001). There was no correlation of miR-29a expression with miR-146a expression, glucose levels, insulin, HbA1c, C-peptide, blood lipids, microalbuminuria and HOMA-IR, HOMA-S, HOMA-β indices (Table 5).

MiR-146a expression was negatively correlated with microalbuminuria (r = -0.21; *P*=0.03) and miR-147b (r = -0.29; *P*=0.003) in patients with T2DM. However, no correlation between the expression levels of miR-146a with the several parameters such as the levels of glucose, insulin, HbA1c, C-peptide, blood lipids, and HOMA-IR, HOMA-S, HOMA-β indices (Table 4). In the newly diagnosed T2DM patients who have not yet received treatment, the expression level of miR-146a correlated with the triglyceride levels (r = 0.49; P < 0.001) and correlated with microalbuminuria (r = -0.28; *P* = 0.045); miR-147b expression (r = -0.29; *P* = 0.04) and but was not uncorrelated with insulin, C-peptide, blood lipids, HOMA-IR, HOMA-S, HOMA- β (*P* > 0.05) (Table 5).

In the T2DM group, the expression level of free plasma miR-147b was moderately positively correlated with glucose concentration (r = 0.34; *P* < 0.001) and a low level with HbA1C (r = 0.23; *P* = 0.02), microalbuminuria (r = 0.29; *P* = 0.003), negatively correlated with C-peptide (r = -0.21; *P* = 0.03) and HOMA-β (r = -0.35; *P* < 0.001), but not correlated with insulin, blood lipids, HOMA-IR, HOMA-S, (*P* > 0.05) (Table 4). For the group of newly diagnosed T2DM patients who received no treatment for the first time, the expression level of free plasma miR-147b was moderately positively correlated with blood glucose concentration (r = 0.48; *P* < 0.001), inversely correlated with C-peptide concentration (r = -0.38; *P* = 0.006) and with HOMA-β (r = -0.52; *P* < 0.001), however, we did not find any correlation with insulin, HbA1C, blood lipids, microalbuminuria, HOMA-IR and HOMA-S (*P* > 0.05) (Table 5).

### The association of expression levels of miR-29a, miR-146a, and miR-147b with clinical parameters

We analyzed the relationship between the expression levels of miR-29a, miR-146a and miR-147b with clinical and subclinical features of patients with T2DM and observed that the expression levels of miR-29a in the groups with and without microalbuminuria were not significantly different (*P* > 0.05). The expression levels of miR-146a were lower while the miR-147b expression levels were higher in the group of T2DM patients with microalbuminuria compared to that without microalbuminuria (*P* < 0.05). Furthermore, the expression levels of miR-29a, miR-146a, and miR-147b in patients with T2DM were not significantly different the overweight and non-overweight patients, between patients with and without hypertension, as well as between patients with and without metabolic syndromes (*P* > 0.05) (Table 6).

### Diagnostic value of miR-29a, miR-146a, and miR-147b

**Figure 2:**
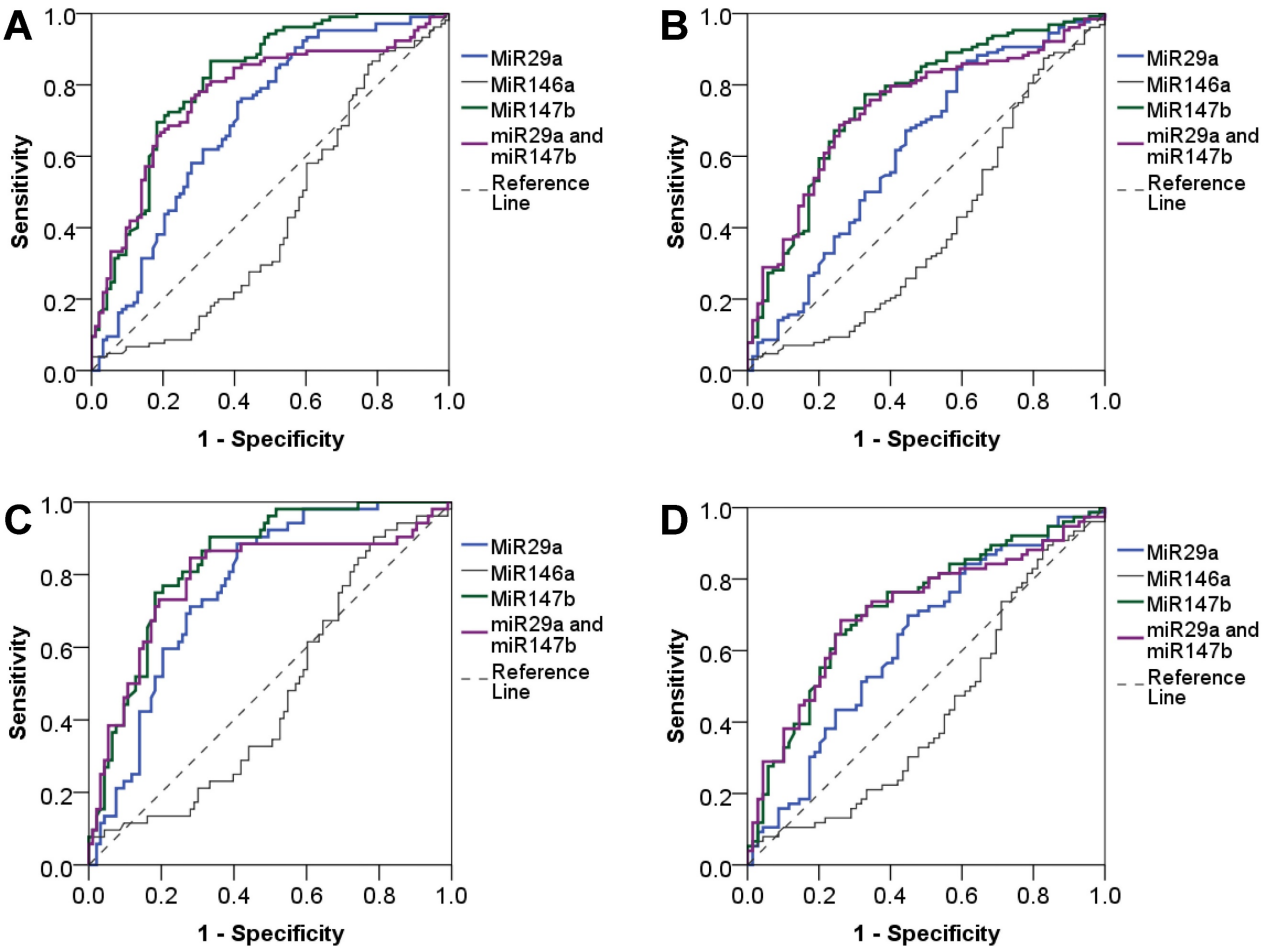
** Diagnostic value of plasma miR-29a, miR146a and miR-147b expression levels.** Diagnostic performance of plasma miR-29a, miR146a and miR-147b expression levels as well as the combination of miR-29a and miR-147b to detect T2DM (**A**), and to detect beta cell dysfunction in T2DM patients (**B**), to detect newly diagnosed T2DM (**C**), and to detect beta cell dysfunction in patients with newly diagnosed T2DM (**D**).

We evaluated the diagnostic value of miRNAs using the area under the ROC curve (AUC) and found that miR-29a and miR-147b showed a good diagnostic performance to identify T2DM (AUC = 0.70 and 0.81, respectively). When combined miR-29a and miR-147b, the diagnostic performance was not increased (AUC = 0.77). The diagnostic value of miR-29a and miR-147b to discriminate individuals with reduced beta cell function was also practical (AUC = 0.62 and 0.75, respectively) while miR-146a had a poor diagnostic performance. MiR-146a had a low diagnostic value for discriminating T2DM and beta cell dysfunction. For the detection of newly diagnosed T2DM patients, miR-29a and miR-147b, as well as the combination of miR-29a and miR-147b showed a great diagnostic value (AUC = 0.77, 0.84 and 0.79, respectively). MiR-29a and miR-147b, and the combination of miR-29a and miR-147b also could discriminate individuals with reduced beta cell function (AUC = 0.63, 0.72 and 0.71, respectively). However, miR-146a has no diagnostic value for T2DM and beta cell dysfunction (Figure 2). In addition, we observed that miR-29a, miR-146a, and miR-147b expression had no diagnostic value for insulin resistance (data not shown).

## Discussion

The pathogenesis of T2DM is a complex process with the involvement of multiple mechanisms and factors. MiRNAs have important roles in maintaining homeostasis in glucose and lipid metabolism, pancreatic beta cell development, and insulin production and secretion. Alterations in miRNAs expression are associated with the early onset of the disease, severity, clinical outcomes and complications of T2DM 18. Our study showed that the expression of miR-29a in the disease group (both untreated and treated groups of patients with T2DM) was significantly higher than that in the control group. This finding is similar to a recent study showing the overexpression of miR-29a in the musculoskeletal system of humans and mice with T2DM, thereby inhibiting the glucose absorption and glucose metabolism effects of insulin 19. Similarly, overexpression of miR-29a interfered with the effect of insulin in the regulation of blood glucose in pancreatic beta cells. Notably, reducing the expression of miR-29a improved beta cell function 8. Our results are also in line with a previous study showing that the expression of miR-29a was higher in the serum of patients with T2DM than in the control group 20. This suggests that miR-29a is associated with the pathogenesis of T2DM. Similar to miR-29a, miR-147b expression is significantly higher in patients with T2DM than in the control group. We also found similar results when we analyzed the subjects with newly diagnosed T2DM and treated T2DM. However, there was no difference in the expression level of miR-147b in the newly diagnosed T2DM group and the treated T2DM group. This result suggested that the expression of miR-147b is not affected by the drug and the duration of T2DM.

The expression of mir-146a in the group of T2DM and the newly diagnosed T2DM does not differ from that in the control group, however, the expression of this miRNA is still a controversial issue. Several studies found that miR-146a expression was significantly reduced in patients with T2DM. Particularly, a study in Mexico (2019) showed that the expression of miR-146a was lower in the pre-diabetes and T2DM groups and there was no relationship between miR-146a expression and beta cell function 21. MiR-146a is considered an anti-inflammatory factor and circulating miR-146a levels are decreased in patients with T2DM. MiR-146a participates in the mechanism of insulin resistance in the pathogenesis of diabetes 22. However, another study showed an increased miR-146a expression in the patient group compared to controls 23. A reason could be that the rs2910164 polymorphism of miR-146a leads to an unstable conformation of pre-miR-146a 24. Further studies are needed to evaluate the expression level as well as the role of miR-146a in patients with T2DM.

Similar to a previous study 25, we found an inverse correlation between the expression level of miR-29a and the duration of disease and the expression of miR-29a in the newly diagnosed T2DM group was higher than in the treated group. These results imply that treatment of the T2DM reduces miR-29a expression over time and not time per-se. There was a positive correlation of miR-29a expression with fasting blood glucose and HbA1C, which are similar to the results of a previous study suggesting that inhibiting the expression of miR-29a improved beta cell function 26. For miR-146a, no correlation between miR-146a expression and blood glucose levels of patients with T2DM and the newly diagnosed T2DM group without treatment was observed. In our study, the expression of miR-147b was positively correlated with blood glucose level. More interestingly, we noted an inverse correlation of miR-147b expression with C-peptide and HOMA-β levels. There is a correlation of free plasma miR-147b expression with blood glucose level. It seems that the overexpression of miR-147b inhibits beta cells to decrease blood insulin causing hyperglycemia. Further studies are needed in patients with T2DM to confirm this mechanism.

Although no correlation between the expression of miR-29a and miR-147b with the patient's blood lipid levels was observed, there is a correlation between miR-146a and blood triglyceride levels in patients with newly diagnosed T2DM and all patients with T2DM. Our study is similar to the study showing no correlation between miR-146a expression and blood lipids 22. A study by Huang et al. (2016) showed that plasma miR-29a was positively correlated with LDL-c levels 27. We found that the expression of miR-146a was lower in the group of T2DM with microalbuminuria and was negatively correlated with microalbuminuria levels. When evaluating subjects with the newly diagnosed T2DM group, we also noted this correlation. For miR-147b, we found that the expression was higher in the group of T2DM with microalbuminuria and had a positive correlation with the concentration of microalbumin in the urine. A recent study in patients with T2DM with renal complications showed that plasma miR-29a expression decreased in the group with macroalbuminuria compared with the group with microalbuminuria and the control group 25. The role of miR-146a in diabetic nephropathy has also been investigated. MiR-146a has a protective effect on the kidneys in the early stages of diabetic nephropathy through the inhibition of inflammatory genes. Loss of this protective mechanism accelerates diabetic nephropathy 28. Therefore, different miRNAs may have different roles in kidney damage. Our study contributes to confirming the role of miR-146a in kidney disease in patients with T2DM.

Insulin resistance is an important factor involved in the pathogenesis of T2DM, which often precedes the onset of T2DM by 5 to 10 years. To evaluate the role of miRNAs in the insulin resistance mechanism in T2DM, we analyzed the relationship between the expression of free plasma miRNAs and the insulin resistance (HOMA-IR) and insulin sensitivity (HOMA-S) indices. The results showed that miR-29a, miR-146a and miR-147b were not correlated with HOMA-IR and HOMA-S.

The role of miRNAs in the pathogenesis of diabetes has been well documented and is considered a potential biomarker for the diagnosis and prognosis of T2DM. An inverse correlation of miR-29a expression with the HOMA-β suggests that miR-29a expression is related to beta cell function. In pancreatic cells, high glucose levels stimulate mitochondrial oxidation, which increases the intracellular ATP/ADP ratio, thereby promoting insulin secretion by beta cells with the involvement of the gene monocarboxylate transporter-1 (*MCT1*). Therefore, the abnormal expression of *MCT1* affects insulin secretion. All three isoforms of miR-29 including miR-29a, miR-29b, and miR-29c were highly expressed in pancreatic beta cells and act on the promoter region of the *MCT1* gene and inhibit their expression hence preventing insulin secretion by pancreatic beta cells. High blood glucose levels increased the expression of miR-29a, thereby inhibiting the insulin secretory function of INS-1E beta cells. We found similar results that the expression of miR-147b was negatively correlated with C-peptide concentration and HOMA-β in patients with T2DM as well as in newly diagnosed T2DM. Unlike miR-29a and miR-147b, our results found no correlation between miR-146a expression and beta cell function indicators (C-peptide, HOMA-β) in the patient group as well as in the newly diagnosed T2DM patients, which are consistent with a previous observation 23. Importantly, we evaluated the diagnostic value of miRNAs in T2DM and beta-cell dysfunction. The results showed a good diagnostic performance of T2DM and beta-cell dysfunction with miR-147b and miR-29a while miR-146a showed no diagnostic value for T2DM and beta-cell functional impairment. Thus, this result suggests that only miR-147b could be a potential biomarker for the diagnosis of pancreatic beta cell dysfunction in patients with T2DM.

In conclusion, the results of our study showed that the plasma miR-29a and miR-147b expression levels in T2DM patients are significantly associated with T2DM while miR-146a shows poor evidence in relation to T2DM. MiR-147b shows potential as a biomarker for the diagnosis of T2DM and pancreatic beta cell dysfunction.

## Figures and Tables

**Table 1:** Primers used for cDNA synthesis and quantitation of microRNAs

Studied miR	Mirbase Accession code	Stem-loop primer	Forward primer
Cel-miR-39	MIMAT0000010	TGTGTTTCACCGGGTGTAAATC	gtgcagggtccgaggt
Mir 29a	MIMAT0000086	TGGGGTAGCACCATCTGAAAT	gtgcagggtccgaggt
Mir 146a	MIMAT0000421	GGTGTGCCTCTGAAATTCAGTT	gtgcagggtccgaggt
Mir 147b	MIMAT0000222	GTGTTGTGTGCGGAAATGC	gtgcagggtccgaggt

**Table 2:** Characteristics of patients with type 2 diabetes mellitus and controls

Characteristics	All patients with T2DM (n=105)	Control group (n=93)	*P* value (*)	Patients with newly diagnosed T2DM (n=52)	Patients with treated T2DM (n=53)	*P* value (**)
***Demographics***						
Age (years)	55 (39-70)	55 (34-70)	NS	54.5 (39-69)	58 (43-70)	0.029
Gender (M/F)	52/53	38/55	NS	29/23	23/30	NS
***Anthropometrics***						
WHR	0.92 (0.8-1.04)	0.89 (0.76-1.1)	< 0.001	0.9 (0.81-1.02)	0.92 (0.8-1.04)	NS
Height (m)	1.58 (1.42-1.74)	1.58 (1.45-1.78)	NS	1.6 (1.45-1.73)	1.57 (1.42-1.74)	NS
Weight (kg)	55 (35-85)	57 (36-84)	NS	54 (37-80)	61 (35-85)	0.014
BMI	23.37 (15.22-30.85)	22.94 (13.38-33.27)	NS	22.39 (15.22-30.47)	24.39 (15.98-30.85)	< 0.0001
***Biochemical and clinical characteristics***						
Glucose (mmol/L)	8.9 (5.8-16.7)	5.3 (4.6-5.6)	< 0.0001	10.35 (7.2-16.7)	7.5 (5.8-12.6)	< 0.0001
Glucose 2h (mmol/L)	20.35 (10.2-47.4)	6.6 (5.5-7.7)	< 0.0001	20.55 (10.2-47.4)	17.55 (16.7-18.4)	NS
AST (U/L)	28.1 (13.9-226)	28.7 (17-704.4)	NS	28.25 (13.9-190.4)	27.8 (15.8-226)	NS
ALT (U/L)	33.1 (12.4-198.4)	30.4 (12.9-743.7)	0.021	32.9 (17.4-198.4)	33.1 (12.4-84.8)	NS
Total bilirubin (µmol/L)	10.15 (5.4-30.4)	13.3 (8.4-28.8)	NS	9.35 (5.4-30.4)	11.95 (5.4-24)	0.002
Direct bilirubin (µmol/L)	3.8 (1.4-19.6)	3.9 (1.8-7.7)	NS	3.55 (1.4-19.6)	3.95 (1.5-8.3)	NS
Blood urea (mmol/L)	4.9 (1.1-8.9)	5 (2.3-11.7)	NS	4.95 (1.1-8.9)	4.7 (3.1-8.1)	NS
Blood creatinine (µmol/L)	61.9 (32.5-130.4)	57.1 (37.3-97.1)	NS	63.2 (32.5-130.4)	60.2 (33.5-106.6)	NS
Triglycerides (mmol/L)	1.87 (0.68-10)	1.56 (0.51-17.87)	0.032	1.97 (0.78-10)	1.83 (0.68-7.95)	NS
Total cholesterol (mmol/L)	5.35 (2.54-10.05)	5.38 (2.32-9.02)	NS	5.68 (2.54-10.05)	4.8 (3.23-7.53)	< 0.0001
HDL-C (mmol/L)	1.3 (0.5-2.07)	1.26 (0.68-2.8)	NS	1.35 (0.5-1.95)	1.27 (0.74-2.07)	NS
LDL-C (mmol/L)	3 (0.6-6.3)	3.2 (0.01-6.2)	NS	3.25 (0.6-6.3)	2.5 (0.6-4.9)	0.001
Glycosylated hemoglobin (HbA1c) (%)	7.6 (4.7-14.3)	5.4 (4.5-6)	< 0.0001	8.6 (7.1-14.3)	6.8 (4.7-9.7)	< 0.0001
Insulin (µU/mL)	9.5 (1.5-33.62)	8.9 (2.98-39.59)	NS	8.61 (1.5-24.57)	10.24 (2.4-33.62)	NS
HOMA-IR	1.38 (0.55-3.52)	1.46 (0.84-2.6)	NS	1.44 (0.89-3.52)	1.35 (0.55-2.53)	NS
HOMA-β	39.6 (7.9-98.8)	112.6 (66.7-180.7)	< 0.0001	29.9 (7.9-73.1)	50.1 (15.6-98.8)	< 0.0001
QUICKI	72.7 (28.4-182.5)	68.7 (38.5-119.4)	NS	69.4 (28.4-112.9)	74.3 (39.6-182.5)	NS
C-peptide (ng/mL)	1.64 (0.68-3.45)	1.96 (1.12-3.46)	0.026	1.48 (0.8-3.45)	1.67 (0.68-2.96)	NS
Protein (ng/mL)	69.4 (55.6-88.1)	67.9 (63-86.6)	NS	69.1 (55.6-83.7)	73.1 (58.3-88.1)	NS
Albumin (ng/mL)	40.4 (26.2-52.5)	43.8 (39.3-46.6)	NS	38.1 (27.4-52.5)	42.2 (26.2-49.5)	0.001
Microalbuminuria (mg/24h)	30 (10-5000)	10 (10-30)	< 0.0001	30 (10-1000)	30 (10-5000)	NS
Red blood cells (G/L)	5.1 (3.76-7.75)	4.89 (4.02-6.45)	NS	5.04 (3.76-7.75)	5.19 (4.31-7.7)	0.015
Hemoglobin (mg/mL)	15.15 (10.2-21.3)	14.9 (10.7-18.3)	NS	14.9 (10.2-17)	15.3 (11.9-21.3)	NS
Hematocrit (%)	45.55 (33.6-62.7)	45.1 (35.2-56.8)	NS	45 (33.6-53.2)	45.9 (36.3-62.7)	0.043
White blood cells (G/L)	7.1 (4-12.4)	7.4 (3.8-12.7)	NS	6.7 (4-12.4)	7.1 (4.3-12.4)	NS
Neutrophils (G/L)	55.45 (2.5-79.3)	51.7 (3.6-71)	NS	7.1 (2.5-66.5)	59.8 (50.4-79.3)	< 0.0001
Lymphocytes (G/L)	33.35 (1.5-46.3)	38.6 (1.9-54.7)	< 0.0001	4.1 (1.5-46.3)	34.1 (15.7-44.7)	0.003
Monocytes (G/L)	5.4 (0.2-20)	7.5 (0.3-19.6)	0.003	0.75 (0.2-20)	6 (2.7-11.6)	0.006
Platelets (G/L)	210 (85-516)	188 (108-1891)	NS	202 (145-317)	217 (85-516)	NS

T2DM: type 2 diabetes mellitus; BMI: Body mass index; WHR: waist-to-hip ratio; AST: A*spartate aminotransferas* ; ALT: Alanine *aminotransferase* ; HDL-C: high-density lipoprotein - cholesterol; LDL-C: low-density lipoprotein - cholesterol; HOMA-IR: Homeostasis Model Assessment -Insulin Resistance; QUICKI: Quantitative insulin Sensitivity Check Index; HOMA-β: homeostatic model assessment- β-cell function; NS: not significant; NA: not applicable; Values given are medians and range; (*) Comparison between patients with T2DM and control individuals; (**) Comparison between Patients with newly diagnosed T2DM and patients with treated T2DM.

**Table 3:** Correlation of miR-29a, miR146a and miR-147b expression levels with clinical characteristics in patients with T2DM

Characteristics	MiR-29a		MiR-146a		MiR-147b	
	Correlation coefficient (r)	*P* value	Correlation coefficient (r)	*P* value	Correlation coefficient (r)	*P* value
Age (years)	0.06	0.52	-0.2	0.04	0.11	0.42
Disease duration (years)	**-0.28**	**0.004**	-0.17	0.09	0.03	0.83
Height (m)	0.059	0.55	0.092	0.35	-0.04	0.77
Weight (kg)	-0.07	0.46	0.047	0.64	-0.03	0.75
Body mass index (BMI) (kg/m^2^)	-0.12	0.22	0.007	0.95	-0.08	0.55
Waist-to-Hip Ratio (WRH)	-0.064	0.52	-0.1	0.31	-0.002	0.99
Ventricular blood pressure (mmHg)	-0.065	0.51	0.05	0.63	0.1	0.33
Diastolic blood pressure (mmHg)	-0.022	0.83	0.09	0.34	-0.02	0.83

**Table 4:** Correlation of miRNAs expression levels with laboratory parameters, HOMA in patients with T2DM

Parameters	miR-29a		miR-146a		miR-147b	
	Correlation coefficient (r)	*P* value	Correlation coefficient (r)	*P* value	Correlation coefficient (r)	*P* value
**Glucose (mmol/l)**	**0.39**	**< 0.001**	0.014	0.89	**0.34**	**< 0.001**
**HbA1C (%)**	**0.33**	**0.001**	0.04	0.71	**0.23**	**0.02**
Insulin (%)	0.06	0.52	-0.015	0.88	0.04	0.7
C-peptide (ng/ml)	-0.12	0.22	-0.01	0.89	**-0.21**	**0.03**
HOMA-IR	-0.016	0.87	-0.02	0.82	-0.09	0.36
**HOMA-β**	**-0.35**	**< 0.001**	-0.03	0.77	**-0.35**	**< 0.001**
TC (mmol/l)	-0.023	0.81	0.05	0.6	-0.01	0.91
Triglycerid (mm/l)	0.16	0.12	**0.22**	**0.03**	-0.07	0.94
HDL (mmol/l)	0.1	0.32	-0.019	0.84	0.1	0.3
LDL (mmol/l)	-0.027	0.78	-0.04	0.67	-0.005	0.96
Microalbuminuria	0.16	0.11	**-0.21**	**0.03**	**0.29**	**0.003**
**MiR-146a**	**0.2**	**0.045**				
**MiR-147b**	**0.47**	**< 0.001**	**-0.29**	**0.003**		

T2DM: type 2 diabetes mellitus; HDL-C: high-density lipoprotein - cholesterol; LDL-C: low-density lipoprotein - cholesterol; HOMA-IR: Homeostasis Model Assessment -Insulin Resistance; QUICKI: Quantitative insulin Sensitivity Check Index; HOMA-β: homeostatic model assessment- β-cell function.

**Table 5:** Correlation of miRNAs expression levels with laboratory parameters, HOMA in patients with newly diagnosed T2DM

Parameter	miR-29a		miR-146a		miR-147b	
	Correlation coefficient (r)	*P* value	Correlation coefficient (r)	*P* value	Correlation coefficient (r)	*P* value
**Glucose (mmol/l)**	0.03	0.83	-0.15	0.31	**0.48**	**< 0.001**
**HbA1C (%)**	0.024	0.87	0.022	0.88	0.14	0.34
Insulin (%)	-0.13	0.73	0.001	0.99	-0.17	0.24
C-peptide (ng/ml)	-0.26	0.07	0.14	0.32	**-0.38**	**0.006**
HOMA-IR	-0.268	0.055	0.08	0.56	-0.18	0.21
**HOMA-β**	-0.12	0.39	0.16	0.24	**-0.52**	**< 0.001**
TC (mmol/l)	-0.06	0.69	0.02	0.9	-0.02	0.9
Triglycerid (mm/l)	0.06	0.67	**0.49**	**< 0.001**	0.1	0.46
HDL (mmol/l)	0.06	0.65	-0.24	0.09	0.08	0.56
LDL (mmol/l)	0.02	0.89	-0.2	0.15	0.13	0.37
Microalbuminuria	-0.07	0.6	**-0.28**	**0.045**	0.13	0.37
**MiR-146a**	0.26	0.06				
**MiR-147b**	**0.45**	**0.001**	**-0.29**	**0.04**		

**Table 6:** The association of expression levels of miR-29a, miR-146a, and miR-147b with clinical parameters

miRNAs expression levels (2^-Delta Ct)	Overweight			Hypertension			Metabolic syndrome			Microalbuminuria		
	Yes (n=49) Median (25%-75%)	No (n=49) Median (25%-75%)	*P* value	Yes (n=50) Median (25%-75%)	No (n=55) Median (25%-75%)	*P* value	Yes (n=72) Median (25%-75%)	No (n=33) Median (25%-75%)	*P* value	Yes (n=75) Median (25%-75%)	No (n=30) Median (25%-75%)	*P* value
**MiR-29a (x10^-4^)**	0.17(0.075-0.37)	0.25(0.1-0.48)	> 0.05	0.18(0.07-0.44)	0.19(0.1-0.46)	> 0.05	0.17(0.07-0.41)	0.26(0.12-0.48)	> 0.05	0.2(0.1-0.52)	0.19(0.08-0.37)	> 0.05
**MiR-146a (x10^-4^)**	2(0.29-15)	1.9(0.24-8.4)	> 0.05	3.28(0.34-23.6)	1.93(0.18-6.1)	> 0.05	1.93(0.28-12.8)	2.77(0.23-10.87)	> 0.05	**1.2(0.07-4.4)**	**2.9(0.4 - 25)**	**< 0.05**
**MiR-147b (x10^-4^)**	14(5.1-35)	24(8.4-52)	> 0.05	16.49(5.04-53.64)	18.48(7.93-39.61)	> 0.05	16.49(5.55-39.75)	24.05(7.1-43.96)	> 0.05	**29(14 - 92)**	**14(5.8 - 37)**	**< 0.05**
